# Association of urinary albumin-to-creatinine ratio with lipid abnormalities and glycemic control in patients with type 2 diabetes mellitus

**DOI:** 10.1097/MS9.0000000000001045

**Published:** 2023-07-19

**Authors:** Sitaram Khadka, Gopal K. Yadav, Prativa Subedi, Kapil Amgain, Arun Sharma, Rinku Joshi

**Affiliations:** a Shree Birendra Hospital, Nepalese Army Institute of Health Sciences; b Nepal Health Research Council, Kathmandu; c Narayani Sub-Regional Hospital, Birgunj; d Karnali Academy of Health Sciences, Jumla, Nepal

**Keywords:** albuminuria, dyslipidemia, proteinuria, urinary albumin creatinine ratio

## Abstract

**Introduction:**

While proteinuria aggravates dyslipidemia in diabetic patients, dyslipidemia further worsens proteinuria via inflammatory cytokines-mediated glomerular damage. Urinary albumin creatinine ratio (ACR) is an easy and reliable method of detecting proteinuria. This study aims to determine the association of ACR with lipid abnormalities and glycemic control in the Nepalese population.

**Methods:**

This was a cross-sectional study conducted among 201 diabetes patients visiting the outpatient department of internal medicine. Based on ACR values, patients were categorized as nonalbuminuric (less than 300 μg/mg) or albuminuric (more than 300 μg/mg). An unpaired *t*-test was used to compare the mean of various lipoproteins in these two categories. Binary logistic regression was used to check the association of ACR with sociodemographic factors (age, sex, and education), hypertension, and glycated hemoglobin.

**Results:**

Albuminuric patients had higher mean cholesterol (192.8±53.5 vs. 184.2± 37.6; *P*=0.209), triglyceride (194.9±97.8 vs. 164.4±73.7; *P*=0.017) and low-density lipoprotein (99.9±38.4 vs. 90.0±27.4; *P*=0.034) but lower high-density lipoprotein (53.9±18.5 vs. 61.3±19.9; *P*=0.008) compared to nonalbuminuric patients. There was a significant difference in mean HbA1c values across albuminuria and nonalbuminuria groups (7.1±1.1 vs. 6.7±0.8; OR: 1.4, 95% CI=1.1–1.9, *P*=0.030).

**Conclusions:**

Urine ACR of more than 30 mg/gram was associated with higher triglyceride and low-density lipoprotein levels and lower high-density lipoprotein levels. The HbA1c level strongly correlates with the development of albuminuria.

## Introduction

Highlights

- Urine albumin creatinine ratio greater than 30 mg/g was associated with higher triglyceride and low-density lipoprotein levels but lower high-density lipoprotein levels.
- Age, sex, education, and hypertensive status were not significantly associated with the albumin creatinine ratio.
- There were no significant differences in the mean value of total cholesterol across albuminuric and nonalbuminuric groups.
- The HbA1c level strongly correlates with the development of albuminuria.

Microalbuminuria is an early marker of diabetic nephropathy^1^. Estimation of albuminuria via 24 h urinary collection was initially considered the gold standard^2^. However, it is quite time-consuming and prone to errors during sample collection. Urine albumin creatinine ratio (ACR) is a quick and convenient method of detecting albuminuria and is measured as a ratio of urinary albumin in micrograms per deciliter (μg/dl) and serum creatinine in milligrams per deciliter (mg/dl)^3^. American Diabetes Association and National Kidney Foundation have defined a cut-off value of 30–300 μg/mg and more than 300 μg/mg for microalbuminuria and macroalbuminuria, respectively^4^. ACR values can be best predicted using the first, early morning void sample^5^. However, random untimed ACR values can also be used for screening purposes, especially in outpatient settings.

Diabetes mellitus (DM) is also associated with dyslipidemia and atherosclerosis^6^, which contribute to the macrovascular complications in diabetes. Diabetic nephropathy and dyslipidemia are interlinked. Dyslipidemia is exacerbated in diabetic patients in the presence of diabetic nephropathy^7^. Studies show an increment in atherogenic lipoproteins like low-density lipoprotein (LDL) and intermediate-density lipoprotein and a reduction in nonatherogenic lipoprotein like high-density lipoprotein (HDL) in type 2 DM patients with diabetic nephropathy^8–10^. Dyslipidemia is thought to worsen with the progression of diabetic nephropathy as the glycated lipoproteins induce glomerular and tubulointerstitial damage via the production of inflammatory cytokines and reactive oxygen species^7–11^. Thus, lipid-lowering therapies are considered to have a crucial role in reducing the progression of diabetic nephropathy^12^. This study aims to determine the association of ACR with lipid abnormalities in type 2 DM in the Nepalese population. It also aims to study the relation of albuminuria with demographic characteristics and glycemic control.

## Methods

### Study design and population

This cross-sectional study was conducted among patients with type 2 DM patients visiting the outpatient Department of Internal Medicine of a tertiary care hospital (Shree Birendra Hospital) from 1 November 2020 to 30 May 2021. Assuming the prevalence of 50%, with a significance level set at 5%, a margin of error of 6.91%, and an infinite population correction, the sample size was calculated to be 201. We included patients above 18 years of age under antidiabetes medications or recently diagnosed with diabetes based on ADA criteria^13^. Diabetic patients requiring insulin within the first year of diagnosis, individuals with a history of diabetic ketoacidosis, and chronic kidney disease (stages 3, 4, and 5) were excluded. We also excluded patients who were already on lipid-lowering drugs, had a family history of dyslipidemia, had comorbid conditions that could contribute to dyslipidemia like hypothyroidism, liver disease, and nephrotic syndrome; and patients using drugs like thiazides, oral contraceptive pills, and corticosteroids.

The study was conducted in accordance with the strengthening the reporting of cohort, cross-sectional and case–control studies in surgery (STROCSS) 2021 criteria^14^.

### Data collection

Sociodemographic information of the participants was collected. The random spot urine sample was collected and urinary albumin concentration was calculated in μg/dl. To maximize the reliability of urine ACR, patients were asked to refrain from vigorous exercise in the 24 h prior to the test. A fasting serum sample was collected from the patients. Major lipoproteins including LDL, intermediate-density lipoprotein, HDL, and total cholesterol were calculated using an automated biochemistry analyzer (Beckman Coulter D×C 700 AU) and glycated hemoglobin (HbA1c) was calculated using an automated HbA1c analyzer.

### Ethical approval

Participants were explicitly explained the purpose of the study and associated risks and benefits. They were assured that their participation was entirely voluntary and that they could withdraw at any point of time of the study. Participants were enrolled only after they willingly consented to the study. The study was approved by the Institutional Review Board of the Nepalese Army Institute of Health Sciences and was in accordance with the Declaration of Helsinki.

### Data analysis

Data were entered in Microsoft Excel 2019 v16.0 (Microsoft) and data analysis was done using Statistical Packages for Social Sciences version 21 (IBM SPSS Corporation, Armonk). The categorical data were expressed as frequency and percentages, while continuous data as mean±SD as applicable. Based on ACR values, patients were categorized into either normoalbuminuria (ACR less than 300 μg/mg) or albuminuria (ACR greater than 300 μg/mg). An unpaired *t*-test was used to compare the mean of various lipoproteins in these two categories. Binary logistic regression was used to check the association of ACR with sociodemographic factors (age, sex, education), hypertension, and HbA1c.

## Results

The mean age of the study participants was 58.9 ±13.9 years. One-fifth of the participants (20.9%, 42) were below 50 years. About half of the participants (50.7%, 102) were female. About one-tenth of the participants (11.4%, 23) were illiterate. About one-fourth of the participants (26.9%, 54) had no hypertension (Table 1).

**Table 1:** Background characteristics of the study participants (*N*=201).

SN	Characteristics	Frequency	Percentage (%)
1	Age (years)		
	Mean±SD	58.9±13.9	
	<50	42	20.9
	≥50 years	159	79.1
2	Sex		
	Female	102	50.7
	Male	99	49.3
3	Education		
	Illiterate	23	11.4
	Literate	178	88.6
4	Hypertension		
	No	54	26.9
	Yes	147	73.1

About two-fifth of the participants (41.3%, 83) had albuminuria. The mean value of total cholesterol, LDL-C, HDL-C, and triglycerides were 187.8±44.9, 94.1±32.7, 58.3±19.6, and 176.9±85.6 mg/dl, respectively. The mean HbA1c of the participants was 6.8±1.1% (Table 2).

**Table 2:** Clinical characteristics of the study participants (*N*=201).

SN	Characteristics	Mean±SD
	Urine	
1	Urine ACR^a^ category	
	No	118 (58.7%)
	Yes	83 (41.3%)
	Lipid profile (mg/dl)	
1	Total-C	187.8±44.9
2	LDL-C	94.1±32.7
3	HDL-C	58.3±19.6
4	TG	176.9±85.6
	HbA1c (%)	6.8±1.1

a Urine ACR: two categories viz., No (<30 mg/g; nonalbuminuria) and Yes (≥30 mg/g; albuminuria).

The mean LDL-C was significantly higher among participants with albuminuria than those without albuminuria (99.9±38.4 vs. 90.0±27.4; 95% CI=−19.0–−0.7; *P*=0.034). There was a significant difference in the mean of TG level among participants with albuminuria than those without albuminuria (194.9±97.8 vs. 164.4±73.7; 95% CI= −55.6–−5.4; *P*=0.017). Similarly, there was a significant difference in HDL-C with a lower mean among participants with albuminuria than those without albuminuria (53.9±18.5 vs. 61.3±19.9; 95% CI=1.9–12.9; *P*=0.008). But there was no significant difference in the mean value of total cholesterol across both groups (192.8±53.5 vs. 184.2±37.6; 95% CI=−22.1–4.9; *P*=0.209) (Table 3).

**Table 3:** Association of ACR and lipid profile of the study participants.

SN	Characteristics	Mean±SD	*t*-value	df	95% CI difference	*P*
1	Total-C					
	Nonalbuminuria	184.2±37.6	−1.262	137.3	−22.1, 4.9	0.209
	Albuminuria	192.8±53.5				
2	LDL-C					
	Nonalbuminuria	90.0±27.4	−2.131	199	−19.0, −0.7	**0.034**
	Albuminuria	99.9±38.4				
3	HDL-C					
	Nonalbuminuria	61.3±19.9	2.670	199	1.9, 12.9	**0.008**
	Albuminuria	53.9±18.5				
4	TG					
	Nonalbuminuria	164.4±73.7	−2.405	144.4	−55.6, −5.4	**0.017**
	Albuminuria	194.9±97.8				

Bold values represent statistical significance.

Nonalbuminuria:<30 /g and Albuminuria:≥30 mg/g.

Age, sex, education, and hypertensive status were not significantly associated with ACR status. However, there was a significant difference in mean HbA1c values across albuminuria and no albuminuria groups (7.1±1.1 vs. 6.7±0.8; OR: 1.4, 95% CI=1.1–1.9, *P*=0.030) (Table 4).

**Table 4:** Association of ACR with background characteristics and HbA1c of the study participants.

		ACR		Binary logistic regression	
SN	Characteristics	*N* (%)	A (%)	OR 95% CI	*P*
1	Age (years)				
	<50	23 (54.8)	19 (45.2)	1 (Ref.)	
	≥50	95 (59.7)	64 (40.3)	0.8 (0.4, 1.6)	0.560
2	Sex				
	Female	57 (55.9)	45 (44.1)		
	Male	61 (61.6)	38 (38.4)	0.8 (0.4, 1.4)	0.409
3	Education				
	Illiterate	13 (56.5)	10 (43.5)		
	Literate	105 (59.0)	73 (41.0)	0.9 (0.4, 2.2)	0.821
4	Hypertension				
	No	30 (55.6)	24 (44.4)		
	Yes	88 (59.9)	59 (40.1)	0.8 (0.4, 1.6)	0.583
5	HbA1c	6.7±0.8	7.1±1.1	1.4 (1.1, 1.9)	**0.030**

Bold values represent statistical significance.

## Discussion

The finding of our study showed that 41.3% of diabetic patients had microalbuminuria or macroalbuminuria, which is quite high compared to a similar study done in Singapore (19.9%) and Japan (30.3%)^15,16^. The mean cholesterol and triglyceride level in our study was similar to that in a study done among the diabetic population in central Nepal but the mean HDL level was relatively higher and the LDL level was relatively lower^17^. A hospital-based study done in Pokhara showed that the prevalence of mixed dyslipidemia in the diabetic population was 88.1 and high LDL was the most common single dyslipidemia^18^.

The mean LDL and TG levels were significantly higher in patients with albuminuria, whereas HDL was significantly lower and the difference in cholesterol levels was not significant. Contrary to this, a study in diabetic patients in Taiwan showed that albuminuria was associated with hypercholesterolemia but not with LDL, HDL, and triglyceride levels^19^. A similar study in Korea showed that TG, cholesterol, and LDL levels were significantly high in patients with overt nephropathy while HDL levels did not bear a statistically significant association^20^. Hypertriglyceridemia is the most consistent risk factor for the progression of diabetic nephropathy in most of the studies^21,22^. All the atherogenic lipoproteins including TG, LDL, and cholesterol thought to accelerate atherosclerosis and induce glomerular damage through renal ischemia and oxidative injury^23^.

Our study did not show any significant relationship between age and the presence of albuminuria. A national registry-based large study done in Saudi Arabia showed that the presence of diabetic nephropathy is highest between the age of 45–69 years. The Framingham Heart Study also showed that the risk of developing stage 3 chronic kidney disease increased by 2.36-fold for each 10-year older age^24^. Since the progression of diabetic nephropathy increases with the duration of diabetes, the early age of diagnosis is corroborated by a greater risk of diabetic nephropathy^25^. There was no sex-based difference in terms of albuminuria in our study. There is varied evidence on the relation of albuminuria with sex in the diabetic population. While some studies suggest no gender preponderance in diabetic albuminuria, both male and female predilection has been shown^26^. In a study by Araki *et al*.^27^, the hazard ratio for females for the progression of diabetic nephropathy was found to be 1.67 times as compared to males. On the other hand, the male is considered an independent risk factor for albuminuria in some studies^28,29^. Although hypertension itself is an important risk factor for albuminuria, there was no significant relation between albuminuria and hypertension in our study. One of the possible explanations for it could be due to the fact that our study has not taken into account the duration of hypertension and the degree of blood pressure control. There was a significant difference in HbA1c levels in patients with and without albuminuria. Poor glycemic control is a strong risk factor for both the development and progression of albuminuria^30,31^. The relationship between glycemic control and albuminuria is linked to podocyte injury mediated by advanced glycated end products^32^. A hospital-based study in India showed a strong correlation between microalbuminuria and HbA1c greater than or equal to 7. This highlights the need of proper glycemic control to prevent the progression of diabetic nephropathy.

### Limitations

Since this is a cross-sectional study, the cause-and-effect relationship between lipid abnormalities and urine ACR cannot be established. Dyslipidemia also correlates with obesity and we have not considered BMI in our study. The duration of diabetes and hypertension has not been taken into account.

## Conclusions

A urine ACR of more than 30 mg/g was associated with higher triglyceride and LDL levels and lower HDL levels. Age, sex, and hypertension did not show any association with albuminuria, while HbA1c level strongly correlates with the development of albuminuria.

## Ethical approval

The ethical approval was obtained from the Institutional Review Committee of the Nepalese Army Institute of Health Sciences (IRC-NAIHS) (Reg No: 245). All procedures performed in studies involving human participants were in accordance with the ethical standards of IRC of NAIHS and with the Declaration of Helsinki, 1964, and its later amendments or comparable ethical standards.

## Patient consent

All the participants were made aware of their voluntary participation and confidentiality prior to the data collection. Written informed consent was obtained from the patient for publication and any accompanying images. A copy of the written consent is available for review by the Editor-in-Chief of this journal on request.

## Parental consent for minors

Written informed consent was obtained from the patient’s legal guardian for publication and any accompanying images. A copy of the written consent is available for review by the Editor-in-Chief of this journal on request.

## Sources of funding

This article did not receive any kind of grants.

## Author contribution

S.K.: conceptualization, methodology, investigation, and writing – original draft; G.K.Y.: conceptualization, data curation, formal analysis, writing – original draft; P.S.: investigation, data curation, formal analysis, writing – original draft; K.A.: methodology, visualization, writing – original draft; A.S. and R.J.: project administration, and writing – review and editing.

## Conflicts of interest disclosure

The authors declare that there are no conflicts of interest.

## Research registration unique identification number (UIN)

1. Name of the registry: Research Registry.
2. Unique identifying number or registration ID: Researchregistry9017.
3. Hyperlink to specific registration (must be publicly accessible and will be checked): https://www.researchregistry.com/browse-the-registry#home/registrationdetails/645f0493ab016b002720a5ae/

## Guarantor

Sitaram Khadka, Shree Birendra Hospital, Nepalese Army Institute of Health Sciences, Kathmandu, Nepal. Tel: +977 985 107 7589. E-mail: sitaram.khadka@naihs.edu.np. ORCID: https://orcid.org/0000-0002-0251-3817.

## Data availability statement

The data are available through the corresponding author on reasonable request.

## Provenance and peer review

Not commissioned, externally peer-reviewed.

## Peer review file

All authors agree to the publication of the peer review file for transparency.
